# Serum heat shock protein 90 alpha levels in mild and severe preeclampsia

**DOI:** 10.1590/1806-9282.20241990

**Published:** 2025-07-07

**Authors:** Kübra Hamzaoğlu Canbolat, Abdullah Serdar Açıkgöz, Koray Gök, Abdullah Tüten, Barış Kaya, Tuğba Kolomuç Gayretli, Mustafa Doğan Özçil, Eduard Malik, Onur Güralp

**Affiliations:** 1 Istanbul University-Cerrahpasa, Cerrahpasa Faculty of Medicine, Department of Obstetrics and Gynecology – İstanbul, Turkey.; 2 Basaksehir Cam and Sakura City Hospital, Department of Obstetrics and Gynecology – İstanbul, Turkey.; 3 University of Health Sciences, Etlik Zubeyde Hanim Women's Health Training and Research Hospital, Department of Obstetrics and Gynecology – Ankara, Turkey.; 4 Hatay Mustafa Kemal University, Faculty of Medicine, Department of Obstetrics and Gynecology – Antakya, Turkey.; 5 Carl von Ossietzky University of Oldenburg, University Hospital for Gynecology and Obstetrics, Klinikum Oldenburg – Oldenburg, Germany.; 6 University Hospital Giessen and Marburg, University Hospital for Gynecology and Obstetrics – Giessen, Germany.

**Keywords:** Blood pressure, Nitric oxide synthase, Preeclampsia, HSP90 heat-shock proteins

## Abstract

**OBJECTIVE:**

The aim of the study was to assess serum heat shock protein 90 alpha levels as a marker in preeclampsia.

**METHODS:**

This cross-sectional study was carried out in 2015 at Istanbul Cerrahpasa University, University Hospital for Obstetrics and Gynecology. The mild (n=60) and severe (n=60) preeclampsia groups were compared with healhty, uncomplicated pregnancies, matched for gestational age within ±1 week (n=120). The main inclusion criteria were women between 18 and 40 years old who had a singleton pregnancy. Pregnant women with any known medical diseases (chronic hypertension, renal diseases, inflammatory diseases, or infections), regular contractions with cervical changes, and premature rupture of membranes were excluded from the study.

**RESULTS:**

Median serum heat shock protein 90 alpha was significantly elevated in mild preeclamptic women compared to women in the gestational age-matched control group [3.41 vs. 2.62 ng/mL, p<0.001]. The mean serum heat shock protein 90 alpha levels were significantly elevated in the severe preeclampsia group compared to their gestational age-matched control group [3.94 vs. 2.55 ng/mL, p<0.001]. Mean serum heat shock protein 90 alpha levels were comparable between the mild and severe preeclampsia groups (3.41 vs. 3.94 ng/mL, p=0.407) and mild early-onset and severe early-onset groups (p=0.104). Serum heat shock protein 90 alpha showed a weak positive correlation with both systolic and diastolic arterial blood pressure.

**CONCLUSION:**

Heat shock protein 90 alpha level was elevated in preeclamptic patients. The level of heat shock protein 90 alpha in serum may be suitable for evaluating endothelial dysfunction.

## INTRODUCTION

Heat shock protein 90 (HSP-90) is one of the most extensive proteins in eukaryotic cells. In normal (non-stressful) conditions, it makes up 1–2% of all cellular proteins. HSP-90 is required to mature and activate proteins involved in signal transduction, control of the cell cycle, protein synthesis and degradation, and morphological evolution^1,2^. HSP-90 is found in the cytosol as two isoforms: HSP-90 alpha and beta. While the level of HSP-90 alpha (α) increases under stress conditions, HSP-90 beta (β) is the main protein, and its level is stable^3^. Increased HSP-90 alpha levels indicate that growth factor-mediated tyrosine kinases are stimulated, and cell cycle and tumor progression are accelerated^4–7^.

HSP-90 must interact in a complex with chaperones, which are involved in the stabilization of denatured proteins and the production of new proteins following cellular stress. HSP-90 also binds to hundreds of other proteins, primarily involved in signal transduction^2^. One of these proteins is endothelial nitric oxide synthase (NOS)^1^. Endothelial NOS provides the balance between nitric oxide (NO) and superoxide production^8^. A study on patients with hypertension showed that serum HSP-90 alpha levels were elevated in these patients. This elevation was explained by the compensation due to the decrease in NO levels in these patients, and it was argued that HSP-90 alpha was an early marker of endothelial injury in hypertensive patients^9^.

There are many studies showing impaired endothelial NOS metabolism in preeclampsia (PE)^10^. Serum levels of HSP-90 alpha, which binds to endothelial NOS and functions together, may differ in preeclamptic pregnant women than non-preeclamptic pregnant women. In the present study, we aimed to assess serum HSP-90 alpha levels as a marker in PE.

## METHODS

In 2015, as a part of an earlier clinical trial^11^. our clinic established a depository of serum samples from preeclamptic pregnancies as the study group and women with healthy uncomplicated pregnancies as the control group, who were recruited at Istanbul University, Cerrahpasa Medical Faculty, Department of Ob/Gyn.

The study protocol of the current study was authorized by the Faculty Ethics Committee (04th of April 2014/8879; 11th of March 2019/39712; 83045809/604.01.02). The patients submitted informed consent to participate in our study. Our study design adhered to the Declaration of Helsinki guidelines.

The study's primary outcome, serum HSP-90, was used in a pilot trial to determine the sample size. The serum HSP-90 levels of a small group of pregnant women (10 women with PE, 10 women with uncomplicated pregnancy) were measured (3.79±1.68 ng/mL in the PE group vs. 2.88±1.51 ng/mL in the non-PE group). The α-error was set at 0.05, and the power was established at 0.8, which gave a calculated sample size of 54 for each subgroup. Women who participated in the pilot trial were not included in the main study's statistical analysis.

Every preeclamptic woman's gestational age (GA) was recorded at the time of recruitment (diagnosis). In the control group, we conducted GA matching within ±1 week. The blood samples were taken during the recruitment process.

The main inclusion criteria were women between 18 and 40 years old who had a singleton pregnancy. Pregnant women with any known medical diseases (chronic hypertension, renal diseases, inflammatory diseases, or infections), regular contractions with cervical changes, and premature rupture of membranes were excluded from the study.

PE and its severe form were defined according to the International Federation of Gynecology and Obstetrics (FIGO) 2019 criteria^12^.

The age, body mass index (BMI), pregnancy history, average blood pressure (ABP), GA at recruitment, GA at the time of delivery, complete blood count (CBC), and blood chemistry were retrieved from the patients’ medical records.

Both the PE and control groups had their venous blood samples drawn between 8:00 and 10:00 in the morning before breakfast. At the time of recruiting, the samples were collected in serum tubes and centrifuged at 1,000*g* for 15 min at 2–8°C. Following the manufacturer's guidelines, the serum part was collected and kept at −80°C until quantitative analysis was conducted using the Human HSP-90 ELISA research kit (SL3096Hu, Sunlong Biotech, Hangzhou, China). The detection range was 0.3–20 ng/mL, and the sensitivity was 0.05 ng/mL. For measuring optical density at a wavelength of 450 nm, a standard automated microplate reader from Thermo Scientific (Microplate Reader, MA, USA) was employed. All biochemical parameters were tested in a blinded setting for the examiner.

The data were compiled using the Statistical Package for the Social Sciences (SPSS 20, Chicago, IL, USA). To assess the distribution homogeneity of all continuous variables, the Kolmogorov-Smirnov test was applied. Comparison of the levels of variables with homogeneous distribution between study and control groups was performed using Student's t-test, and the comparison of variables with heterogeneous distribution was made using the Mann-Whitney U test. Homogeneously distributed parameters were shown as mean±standard deviation (SD), and heterogeneously distributed parameters as median (min–max). A Spearman's correlation test was conducted to evaluate the relationships between HSP-90 alpha and other relevant parameters, adjusting for the remaining variables. A p<0.05 was taken as statistically significant.

## RESULTS

The baseline characteristics, including age, BMI, and parity, were similar between the mild PE (n=60) and control (n=60) groups, as shown in Table 1. Median serum HSP-90 alpha was significantly higher in the mild preeclamptic women compared to the women in the GA-matched control group [3.41 (2.74–4.76) ng/mL vs. 2.62 (2.11–3.23) ng/mL, p<0.001].

**Table 1:** Demographic, clinical, and laboratory features of the women with mild preeclampsia and women in the control group matched for mild preeclampsia.

	Mild preeclampsia (n=60)	Control group gestational age matched for mild preeclampsia (n=60)	p	Test
Age (years)	27.8±5.1	26.4±4.6	0.104	t-test
BMI (kg/m^2^)	29.9±5.5	30.29±4.5	0.687	t-test
Parity (n)	0 (0.00–1.00)	1 (0–2)	0.618	MWU
Systolic ABP (mm/Hg)	150 (140–150)	117 (109–125)	**<0.001**	MWU
Diastolic ABP (mm/Hg)	90 (90–100)	75 (70–80)	**<0.001**	MWU
GA at sampling (w)	33.3±4.8	33.5±4.3	0.821	t-test
GA at birth (w)	35.2±4.1	38.3±1.3	**<0.001**	t-test
WBC (10^9^/L)	11.1±2.9	10.8±3.3	0.583	t-test
Platelet count (10^3^/μL)	210±67	212±62	0.913	t-test
AST (IU/L)	18 (14–25)	17.0 (14–20)	0.630	MWU
ALT (IU/L)	14 (12–20)	12.9 (10–16)	0.072	MWU
Creatinine (mg/dL)	0.62±0.10	0.55±0.11	**<0.001**	t-test
Total bilirubin (mg/dL)	0.40±0.17	0.40±0.12	0.773	t-test
Uric acid (mg/dL)	5.40 (4.77–5.97)	4.2 (3.60–4.65)	**<0.001**	MWU
Urea (mg/dL)	16.0 (13.0–20.0)	10.5 (8.0–14.0)	**<0.001**	MWU
HSP-90 (ng/mL)	3.41 (2.74–4.76)	2.62 (2.11–3.23)	**<0.001**	MWU

ABP: arterial blood pressure; ALT: alanine aminotransferase; AST: aspartate aminotransferase; BMI: body mass index; GA: gestational age; HSP-90: heat shock protein 90; WBC: white blood cells. p<0.05 is significant (marked with bold). MWU: Mann-Whitney U test.

The mean levels of serum HSP-90 alpha were significantly higher in the severe PE group than in its GA-matched control group [3.94 (2.89–4.90) ng/mL vs. 2.55 (2.29–3.31) ng/mL, p<0.001] (Table 2).

**Table 2:** Demographic, clinical, and laboratory features of the women with severe preeclampsia and women in the control group matched for severe preeclampsia.

	Severe preeclampsia (n=60)	Control group gestational age matched for severe preeclampsia (n=60)	p	Test
Age (years)	28.3±5.1	27.6±5.2	0.420	t-test
BMI (kg/m^2^)	30.5±4.0	29.1±4.3	0.066	t-test
Parity (n)	0.00 (0.00–2.00)	1 (0–2)	0.618	MWU
Systolic ABP (mm/Hg)	160 (160–170)	110 (105–120)	**<0.001**	MWU
Diastolic ABP (mm/Hg)	110 (100–110)	70 (60–80)	**<0.001**	MWU
GA at sampling (w)	31.4±4.5	32.1±5.0	0.417	t-test
GA at birth (w)	33.4±4.3	38.7±1.3	**<0.001**	t-test
WBC (10^9^/L)	11.7 (9.5–13.5)	10.00 (8.8–11.2)	0.145	MWU
Platelet count (10^3^/μL)	193±68	240±55	**<0.001**	t-test
AST (IU/L)	18 (15–38)	17.00 (14–21)	0.630	MWU
ALT (IU/L)	16 (12–30)	13.50 (12–17)	0.072	MWU
Creatinine (mg/dL)	0.70±0.17	0.52±0.11	**<0.001**	t-test
Total bilirubin (mg/dL)	0.51±0.27	0.42±0.16	0.773	t-test
Uric acid (mg/dL)	6.10 (5.22–6.87)	4.27 (3.50–4.75)	**<0.001**	MWU
Urea (mg/dL)	20.5 (15.2–25.0)	13.5 (9.2–20.0)	**<0.001**	MWU
HSP-90 (ng/mL)	3.94 (2.89–4.90)	2.55 (2.29–3.31)	**<0.001**	MWU

ABP: arterial blood pressure; ALT: alanine aminotransferase; AST: aspartate aminotransferase; BMI: body mass index; GA: gestational age; SD: standard deviation; HSP-90: heat shock protein 90; WBC: white blood cells. p<0.05 is significant (marked with bold). MWU: Mann-Whitney U test.

Mean serum HSP-90 alpha levels were comparable between the mild and severe PE groups [3.41 (2.74–4.76) ng/mL vs. 3.94 (2.89–4.90) ng/mL, p=0.407] and mild early-onset and severe early-onset groups (p=0.104).

Finally, serum HSP-90 alpha showed a weak positive correlation with both systolic and diastolic blood pressure (ABP), as shown in Table 3.

**Table 3:** The partial correlation analysis of heat shock protein 90 and various parameters in all participants.

	HSP-90 (all participants)	
	Correlation coefficient	p
Age	0.002	0.982
BMI	0.004	0.949
Systolic ABP	0.289	**<0.001**
Diastolic ABP	0.268	**<0.001**
GA at sampling	-0.114	0.079
WBC count	-0.113	0.083
Platelet count	-0.102	0.120
Creatinine	0.053	0.417
Total bilirubin	0.017	0.791
Uric acid	0.021	0.749
Urea	-0.013	0.838
AST	-0.109	0.094
ALT	-0.121	0.063

ABP: arterial blood pressure; ALT: alanine aminotransferase; AST: aspartate aminotransferase; BMI: body mass index; GA: gestational age; HSP-90: heat shock protein 90; WBC: white blood cells. p<0.05 is significant (marked with bold). Spearman's correlation test was separately performed for every parameter by controlling for all remaining parameters in the table.

## DISCUSSION

In this study, we found that the serum level of HSP-90 alpha was significantly elevated in preeclamptic pregnant women than healthy pregnant women. Also, we found that this elevation was positively correlated with both systolic and diastolic blood pressure. The level of HSP-90 alpha in serum may be suitable for evaluating endothelial dysfunction.

Although hypertension is mainly a hemodynamic disorder, there is evidence showing that autoimmunity, characterized by inflammation in the kidneys (which suppresses natriuresis), arterial walls (which causes disruption of endothelial vasodilation), and the central nervous system (stimulate sympathetic outflow), is also effective^13,14^. The main HSP, which may play a role in the development of hypertension, is HSP-70. According to the review by Saghafi et al., they stated that HSP-70 has a role in the pathogenesis of PE and that its measurement helps identify preeclamptic pregnant women^15^.

There are studies showing that HSP-90, like HSP-70, plays a role in the development of hypertension, and its level increases during hypertension. The reason for the increase in HSP-90 levels in hypertensive patients has been explained by the effort to overcome the bio-use insufficiency of endothelial NO in these patients^16,17^.

Reactive nitrogen species covalently add NO to cysteine in proteins and peptides and form S-nitrosylation (SNO), which modulates NO functions^18^. While HSP-90 activates endothelial nitric oxide synthase (NOS3), SNO inhibits both HSP-90 and NOS3^18,19,^. In a clinical trial of Zhang et al., it was determined that NOS3 and HSP-90 were less neutralized, and their levels increased in the placenta of severe preeclamptic women. This situation was explained by compensation for the deficiency in NO bioavailability^20^. In the study by Güzel et al.^21^, serum calcyclin (S100A6) and HSP-90 levels were evaluated from the first trimester to the term in healthy and preeclamptic pregnant women. Compared to healthy pregnant women, serum S100A6 levels decreased significantly after the second trimester, while serum HSP-90 levels increased significantly after the third trimester. The authors explained the increase in serum HSP-90 level in the third trimester in these pregnant women not as a cause leading to the clinical onset of PE but as a result of secondary mechanisms (e.g., inflammation)^21^. Apart from the studies investigating the level of HSP-90 in the serum and placenta of preeclamptic pregnant women, in the study conducted by Gu et al., the level of HSP-90 was measured in the umbilical cord of these pregnancies, and the results showed increased superoxide and decreased HSP-90 production in the endothelial cells of the umbilical cord vein. The authors explained this situation with increased endothelial oxidative stress in the fetus and the mother and placenta in preeclamptic pregnant women, and the decreased endothelial HSP-90 expression leading to increased endothelial oxidative stress^22^.

In light of the above studies, it can be said that placental HSP-90 expression will increase in preeclamptic pregnancies when there is enough time, and it will decrease in the mother's serum and umbilical cord regardless of the time of PE development and severity. However, we expect that the increase in HSP-90 expression in the placenta of these pregnant women should occur in the mother's serum as well. The way to better evaluate this situation may be to measure the HSP-90 alpha level in the serum of these pregnant women instead of the HSP-90 level, because HSP-90 alpha is more sensitive to stress than HSP-90. Accordingly, an increase in HSP-90 alpha is expected when there is oxidative stress and maternal systemic inflammation, such as PE. In support of this, Ekambaram et al.^23^ detected increased HSP-90 alpha and decreased heme-oxygenase expression accompanied by increased oxidative stress in cord blood erythrocytes of preeclamptic pregnancies^23^. However, no study has been conducted to evaluate HSP-90 alpha levels in maternal serum and placenta in preeclamptic pregnant women. Our present study showed that the HSP-90 alpha level is elevated in PE, and this elevation was correlated with systolic and diastolic ABP. Although the elevation of HSP-90 alpha was correlated with ABP, its relationship with the severity of PE could not be demonstrated. The reason may be that even if the number of pregnant women in the study was not low, the sample size was insufficient to show the relationship with the severity of PE.

The findings of the trial by Skórzyńska-Dziduszko et al.^9^ confirm the results of the present study. They found elevated serum Hsp-90a levels in new-onset hypertensive overweight or obese patients and characterized this increase as a protective effort to prevent impaired NO bioavailability in the early phase of hypertension. Therefore, they stated that HSP-90 alpha might be a marker of early endothelial dysfunction due to hypertension^9^.

The strengths of the present study are that the number of healthy and preeclamptic pregnant women is sufficient and that it is the first study showing an increase in serum HSP-90 alpha level in preeclamptic pregnant women. The study's limitations are that the HSP-90 alpha level was only evaluated in the serum, not in the placenta and umbilical cord, and oxidative stress markers and NO metabolism were not studied.

## CONCLUSION

In this study, elevated levels of HSP-90 alpha were detected in the serum of preeclamptic pregnant women for the first time. HSP-90 alpha level was elevated in preeclamptic patients. The level of HSP-90 alpha in serum may be suitable for evaluating endothelial dysfunction. However, further comprehensive, large-scale, randomized, prospective studies are needed.

## Data Availability

The datasets generated and/or analyzed during the current study are available from the corresponding author upon reasonable request.
